# Biomechanical viscoelastic properties in leg muscles of people with hemophilia: a case control study

**DOI:** 10.1016/j.rpth.2026.107000

**Published:** 2026-10-01

**Authors:** Marius Brühl, Fabian Tomschi, Andreas Christian Strauss, Joschua Wiese, Pia Möllers, Alexander Schmidt, Thomas Hilberg

**Affiliations:** 1 Department of Sports Medicine, University of Wuppertal, Wuppertal, Germany; 2 Department of Orthopaedics and Trauma Surgery, University of Bonn, Bonn, Germany

**Keywords:** hemarthosis, hemophilia A and B, muscle strength, muscle tonus, myotonometry

## Abstract

**Background:**

People with hemophilia (PwH) are prone to joint bleeds, resulting in hemophilic arthropathy, potentially altering muscle tissue. Although functional and morphologic impairments are documented, little is known about biomechanical muscle properties in PwH.

**Objectives:**

This study investigates muscle stiffness in relaxed and contracted states in the lower limbs of adult PwH and its relationship to muscle strength and joint health.

**Methods:**

In this cross-sectional case-control study, 40 PwH and 20 healthy controls were assessed. Using myotonometry, stiffness of 8 lower limb muscles was measured under relaxed (*S*_*rel*_) and maximum voluntarily contracted conditions (*S*_*con*_). Stiffness increase rate (*SIR*) was calculated. Joint health was assessed via the Hemophilia Joint Health Score and sonography (Hemophilia Early Arthropathy Detection with Ultrasound), and muscle strength was measured using a mobile isometric force gauge. PwH were stratified by lower limb joint impairment (*PwH*_*minor*_ vs *PwH*_*major*_).

**Results:**

*PwH*_*major*_ exhibited significantly increased *S*_*rel*_ in anterior thigh and calf muscles compared with controls and *PwH*_*minor*_ (all *P* ≤ .05, *r* = 0.397-1.010). Under contraction, *PwH*_*major*_ showed reduced *S*_*con*_ in the tibialis anterior (*P* ≤ .05, *r* = 0.511-0.683) and biceps femoris (*P* = .04, *r* = 0.390). The *SIR* was significantly lower in *PwH*_*major*_ across most muscles (all *P* ≤ .05, *r* = 0.380-0.573), indicating impaired muscle contractility. Furthermore, muscle strength correlated negatively with *S*_*rel*_ (*r*_*s*_ = –0.220 to –0.430) and positively with both *S*_*con*_ and *SIR* (*r*_*s*_ = 0.273-0.449). Hemophilia Joint Health Score correlated with myotonometric parameters (*r*_*s*_ = 0.248-0.683), linking joint damage to altered muscle properties.

**Conclusion:**

Advanced hemophilic arthropathy is associated with increased passive stiffness and reduced active muscle response, particularly in stabilizing muscle groups. Myotonometry may serve as a potential supplementary tool for the exploratory assessment of biomechanical muscle status in PwH.

## Essentials

• Hemophilic arthropathy in people with hemophilia (PwH) alters muscle tissue. This case-control study (40 PwH/20 controls) uses myotonometry.
• *PwH*_*major*_ show significantly increased passive stiffness (*S*_*rel*_) in anterior thigh and calf muscles.
• Contracted stiffness (*S*_*con*_) and stiffness increase rate are reduced in *PwH*_*major*_, indicating impaired contractility.
• Muscle strength and Hemophilia Joint Health Score correlate with stiffness, linking joint damage to muscle dysfunction.

## 1 Background

Hemophilia is a hereditary coagulation disorder characterized by a deficiency or functional impairment of specific clotting factors, leading to an increased risk of spontaneous or trauma-induced bleeding, predominantly into joints and, less frequently, into muscle tissue. In people with hemophilia (PwH), joint bleeds (hemarthroses) are typically associated with pain, joint effusion, and restricted range of motion [1]. Recurrent hemarthroses not only elevate the risk of subsequent bleeding episodes but also initiate progressive intra-articular degeneration. Over time, this process can culminate in hemophilic arthropathy (HA), a chronic joint disease marked by structural damage, inflammation, pain, and functional impairment [2]. HA can also negatively affect the surrounding musculature, leading to muscle atrophy, altered muscle activation patterns, and further reductions in physical function [3].

Prior research dealing with lower extremity muscle properties reveals that PwH possess lower strength [4], [5], lower muscle cross-sectional areas and altered pennation angles and fascicle length [6], altered neuromuscular coordination measured via electromyography (EMG) [7], [8], and impaired functional motor performance [4], [9]. EMG primarily measures the electrical activity produced by skeletal muscles during contraction and relaxation, offering insights into the neuromuscular control and recruitment patterns of muscles [10], whereas morphologic muscle architecture is measured via pennation angles and fascicle length [11]. Although these established approaches are crucial, they do not directly assess the biomechanical characteristics of the muscle itself. In contrast, myotonometry bridges this clinical gap by providing a rapid, highly standardized, and cost-effective noninvasive assessment of biomechanical viscoelastic muscle properties. Its added clinical value lies in its ease of application and the immediate, objective feedback it provides regarding the mechanical state of the tissue. This allows clinicians to quantify functional impairments such as pathologic stiffness, which imaging or EMG cannot capture alone, positioning myotonometry as a potential complementary tool that warrants further investigation for monitoring muscle changes and evaluating targeted rehabilitation interventions in PwH. In addition to musculoskeletal conditions, myotonometry has been extensively validated in various clinical and neuromuscular populations, including stroke [12], Parkinson disease [13], and cerebral palsy [14], demonstrating its high sensitivity for quantifying pathologic alterations in passive muscle stiffness and muscular tone.

Muscle stiffness refers to the muscle’s resistance to deformation [15], and increased muscle stiffness in a relaxed state is generally considered pathologic in individuals with, for instance, osteoarthritis (OA), particularly when associated with pain, impaired mobility, or reduced functional capacity [16]. In musculoskeletal disorders (such as OA), multiple pathophysiological mechanisms are involved, including, among others, structural remodeling in muscle, neuromuscular control changes, inflammatory mediators, pain-mediated adaptive stiffening, and tendon and fascial changes [17], [18]. The myotonometric assessment (using the MyotonPRO device; Myoton AS) of muscle stiffness was shown to possess a consistently high intrarater and inter-rater reliability across different muscles in different populations [19]. Several studies have compared the stiffness of soft tissues between healthy individuals and patients with various musculoskeletal disorders, such as low back pain, neck pain, and patellar tendinopathy, to explore the mechanical properties of soft tissues in these populations [20], [21], [22]. It was recently shown that patients with knee OA demonstrate increased stiffness of the vastus lateralis (VL), and higher stiffness was further associated with less functionality in these patients [16]. Although mechanical muscle properties have been explored in general joint degeneration models such as knee OA [20], [21], muscle alterations in PwH are driven by unique hemophilia-specific pathophysiology. Recurrent intra-articular and periarticular bleeding events induce arthrogenic muscle inhibition, progressive muscle atrophy, and altered electromyographic recruitment patterns [3], [6], [7], [8]. However, direct biomechanical assessments of viscoelastic muscle properties in adult PwH remain entirely uninvestigated. Although the impact of HA on joint health is well documented in PwH, the corresponding changes in muscle biomechanics, specifically how advanced HA alters muscle stiffness in both relaxed and contracted states, remain entirely unknown in adults. Although one previous study evaluated resting stiffness exclusively in children [23], the absence of data regarding adult PwH and their dynamic muscle contractility represents a crucial research gap that this study aims to address.

Moreover, measuring these properties under relaxed and voluntarily contracted conditions can provide a comprehensive understanding of muscle function. In a relaxed state, the measurement reflects the passive biomechanical stiffness of the muscle, whereas in a contracted state, the muscle’s ability to generate force and respond to mechanical stress is evaluated. This approach offers insights into how HA affects biomechanical muscle stiffness and performance in 2 functional states. Based on these considerations, the present study aims to evaluate the biomechanical stiffness of the major leg muscles in PwH, compared with healthy control subjects. In particular, this study aims to answer the following research questions: (1) Are there differences in biomechanical muscle stiffness in a relaxed state between differently affected PwH and matched controls? (2) How do the biomechanical muscle properties differ between PwH and controls when measured in a contracted state? (3) How are the biomechanical muscle properties of PwH associated with muscle strength?

Drawing from pathophysiological mechanisms observed in comparable joint disorders such as OA, we hypothesized that (1) advanced HA is generally associated with increased passive resting muscle stiffness due to structural remodeling; (2) dynamic muscle properties during maximum voluntary contraction are altered in PwH, reflecting functional and neuromuscular impairments; and (3) these altered biomechanical parameters correlate with reduced objective isometric muscle strength.

## 2 Methods

### 2.1 Study design

This cross-sectional case-control study follows the STROBE checklist for case-control studies [24]. The study was conducted in accordance with the Declaration of Helsinki, and the study protocol was approved by the local ethics committee of the University of Wuppertal (MS/AE 220729).

### 2.2 Setting

The study period was from March 2023 to January 2024 at the University of Wuppertal Department of Sports Medicine. PwH were recruited via 2 multiday events for PwH held in April and October 2023, and the control participants were recruited throughout the study period via announcements. After giving verbal and written consent, participants completed a medical history questionnaire to assess their eligibility for participation. Afterward, the participants undressed so that their arms and legs were exposed for examination on an examination table.

### 2.3 Participants

A total of 60 male participants were recruited for this study. Of these, 40 were PwH and 20 were healthy controls. All participants were of White/European ethnicity and held German nationality (100%). The sample size used was estimated based on an a priori analysis using G∗Power (version 3.1; Heinrich-Heine-Universität Düsseldorf). Because of a lack of comparable studies in PwH with HA, studies in patients with knee OA were consulted, as both conditions exhibit comparable clinical and functional limitations despite differing pathogenesis. These studies demonstrated differences in stiffness with a large effect size (*d* = 0.82, *d* = 1.14) [16], [25]. Averaging and converting these (*f* = *d*/2) yields *f* = 0.49. A conservative effect size of *f* = 0.45 was chosen and used for the present independent 1-way model with 3 groups and a power of 90%. This calculation resulted in a minimum sample size of 57.

Included were adult people (aged ≥18 years) with mild (FVIII or IX <40%), moderate (FVIII or IX <5%), and severe (FVIII or IX <1%) hemophilia A or B (PwH) and a healthy male control group (*CG*) of similar age and body mass index (BMI). Only men were included in the study, as severe forms of HA affect almost exclusively men. Exclusion criteria were as follows: other bleeding disorders, bleeding events in the last 2 weeks, the presence of inhibitors, neurologic diseases, acute musculoskeletal injuries, musculoskeletal disorders not associated with hemophilia, and, for the *CG*, the presence of musculoskeletal or neurologic disorders.

### 2.4 Myotonometry

To investigate the viscoelastic characteristics (ie, muscle stiffness) of 8 hemophilia-relevant lower limb muscles, the included subjects were examined on both legs using the handheld device “MyotonPRO” (Myoton AS). In theoretical terms, the MyotonPRO provides an estimate of compressive stiffness by measuring the tissue response to a perpendicular mechanical deformation. This methodology differs from ultrasound shear wave elastography, which assesses stiffness via the propagation velocity of shear waves, or displacement torque methods. In prone and supine positions on a massage table with the feet on the table, the defined measuring points were palpated and marked with a skin marker. The measurement points in the supine position were tibialis anterior (TA) muscle at 33% of the distance between the tibial tuberosity and the lateral malleolus [26], rectus femoris (RF) muscle at two-thirds of the distance from the anterior superior iliac spine to the superior pole of the patella [27], vastus medialis (VM) muscle, and VL muscle at the point of muscle belly bulk [28]. In the prone position, measurement points were localized as follows: gastrocnemius lateralis (GL) muscle and gastrocnemius medialis (GM) muscle at the midpoint between the popliteal fossa and the distal muscle-tendon junction [29], biceps femoris (BF) muscle at 50% of the distance between the ischial tuberosity and the lateral femoral epicondyle, and semitendinosus (SM) muscle at 50% of the distance between the ischial tuberosity and the medial femoral epicondyle [30]. All assessment locations are schematically illustrated in Figure 1.

**Figure 1:**
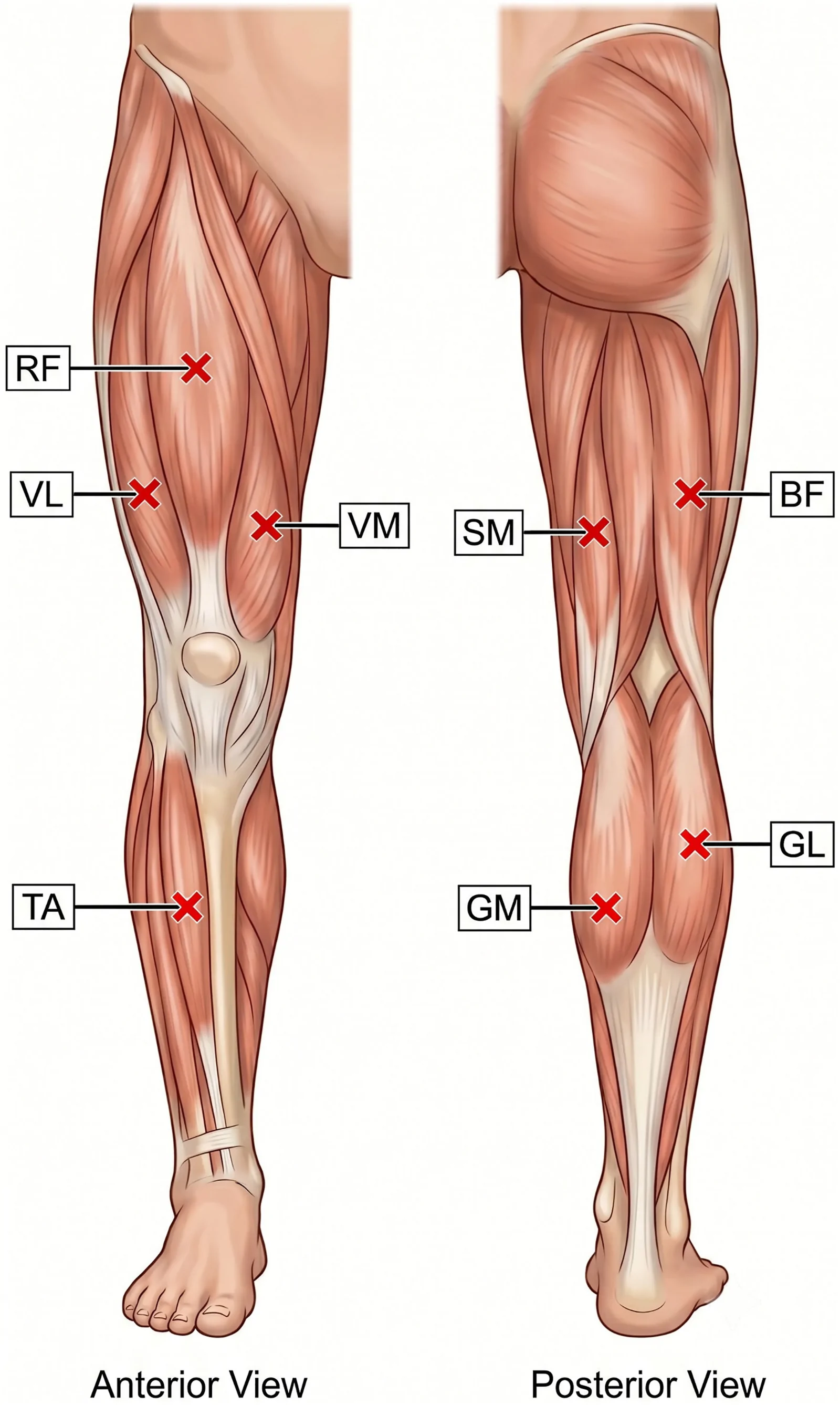
Schematic illustration of the anatomical measurement sites for myotonometric stiffness assessment of lower limb muscles. BF, biceps femoris; GL, gastrocnemius lateralis; GM, gastrocnemius medialis; RF, rectus femoris; SM, semitendinosus; TA, tibialis anterior; VL, vastus lateralis; VM, vastus medialis.

Myotonometry is a noninvasive measurement performed by M.B., an experienced user. This method has been evaluated as reliable in previous studies [19], [31]. Measurements were taken bilaterally, and all data points were pooled for the overall analysis to reflect the general musculoskeletal profile. Care was taken to position the joints as consistently as possible across all participants, as joint positioning significantly affects compressive stiffness [32]. The stiffness refers to the resistance of soft tissues to external forces and is calculated by analyzing damped natural vibrations detected by an integrated accelerometer [19]. The device is equipped with a 3-mm flat probe, which is applied orthogonally to the measuring points with a constant pressure of 0.18 N. The probe then automatically presses on the tissue 5 consecutive times with a frequency of 800 milliseconds and a pressure of 0.4 N for 15 milliseconds. The resulting oscillation of the tissue is recorded using an acceleration sensor with a sampling rate of 3200 Hz [33]. The device internally averages the data from these 5 impulses to calculate the final stiffness value for the respective muscle. The interpretation of the parameter dynamic stiffness (S) in (N/m) to describe the muscle characteristics corresponds to the following formula:

$$S=\frac{a_{\max}\times m_{probe}}{\Delta l}$$

where *a*_*max*_ indicates the maximum acceleration of the damped oscillation, *m*_*probe*_ indicates the mass of the measurement mechanism, and *Δl* indicates the maximum displacement of the tissue [34].

The 8 measurement points were examined in the relaxed muscle condition (*S*_*rel*_), after 5 minutes of relaxation [30], and in the maximally voluntarily contracted condition (*S*_*con*_). Measurements of stiffness of muscles in relaxed and contracted states were shown to be reliable using the MyotonPRO device [33]. In the supine position, RF, VM, and VL were measured, and the participant was instructed to contract the quadriceps as much as possible and to press the back of the knee into the massage position, resulting in knee extension. TA was also measured in the supine position at the maximum possible dorsiflexion of the foot. In the prone position, the participant should let the feet hang over the massage table and perform the maximum plantar flexion for the contraction of GL and GM. BF and SM also took place in the prone position, and the participant performed a knee flexion of 45°, which was limited by a strap against which the participant pressed. The instructions for muscle contraction were based on Lee et al. [33] but had to be adapted for PwH with severe HAs if dorsiflexion of 15°, plantar flexion of 35°, or knee flexion of 90° could not be achieved. In these cases, the maximum reachable angle close to the recommendation was used. The measurement time during which the contraction had to be maintained was 10 seconds [33]. To compare the difference between the stiffness in the maximally voluntarily contracted condition and the stiffness in the relaxed muscle condition, the following formula is used to calculate the stiffness increase rate (*SIR*) [35], with higher values being indicative of better voluntary muscle functioning:

$$SIR=\frac{S_{con}-S_{rel}}{S_{rel}}$$

### 2.5 Clinical joint examination

#### 2.5.1 Hemophilia Joint Health Score

The orthopedic joint status was assessed using the Hemophilia Joint Health Score (HJHS) version 2.1. In this assessment, the ankles, knees, and elbows are systematically assessed for parameters such as axial deformity, muscle atrophy, swelling, range of motion (measured in extension and flexion with a goniometer), crepitus on movement, joint pain in the previous 2 weeks, and muscle strength. The assessment results in a composite score ranging from 0 (indicating optimal joint health) to 124 (indicating severe functional and structural joint impairment) [36].

#### 2.5.2 Hemophilia Early Arthropathy Detection with Ultrasound

The ankle, elbow, and knee joints of PwH were scanned in a standardized manner: synovia, cartilage, and bone defects were documented in a protocol for each joint and in each section. The Philips Lumify handheld ultrasound device was used (Koninklijke Philips N.V.). The “Activity” score describes the hypertrophy of the synovium and ranges from 0 (absent/minimal), 1 (mild/moderate), to 2 (severe). The “Cartilage” score ranges from 0 (normal), 1 (loss of the articular cartilage involving <25% of the target surface), 2 (loss of the articular cartilage involving at least ≤50% of the target surface), 3 (loss of the articular cartilage involving >50% of the target surface), to 4 (complete cartilage destruction or absent visualization of the articular cartilage on the target bony surface), and the “Bone” score ranges from 0 (normal), 1 (mild irregularities of the subchondral bone), to 2 (deranged subchondral bone). Cartilage and Bone are combined into the score “Damage,” and the Global Score sums up Activity and Damage [37].

### 2.5.3 Strength tests

The isometric force of the knee extension and flexion (quadriceps femoris and ischiocrural musculature) was measured using a mobile force gauge (KD 9363 with DMS measuring amplifier GVS-2; ME-Measuring Systems GmbH) while participants were seated. The maximum force was generated over a 5-second period, maintained for 2 seconds, and then released, with the knee positioned at 75° of flexion for knee extension and 30° of knee flexion for flexion force [38]. This procedure was repeated 3 times for each leg, with a rest interval of 45 seconds between trials. In addition, bilateral hand grip strength was assessed using a hand dynamometer (Baseline) in a seated position, also 3 times for each arm, with the elbow flexed to 90°. For knee flexion, knee extension, and hand grip strength tests, the mean of the 3 trials was calculated for each limb.

### 2.5.4 Statistics

PwH were stratified into 2 groups based on the sum of the HJHS of the lower extremities (knee and ankle on both sides). To determine the threshold between groups, the median HJHS of the study cohort (24.0) was used as a cutoff point. This classification yielded the following groups: PwH with a score less than or equal to the median were categorized as the “minorly affected” group (*PwH*_*minor*_), whereas PwH with a score above the median were categorized as the “majorly affected” group (*PwH*_*major*_), as done before [4], [39]. This median split approach was chosen to objectively divide the cohort into 2 equal subgroups, reflecting those with a lower vs higher degree of structural joint arthropathy.

Statistical analyses were performed using the IBM SPSS 29 software (IBM Corp.), and graphs were plotted using GraphPad Prism 10 (GraphPad Software). Because of non-normally distributed data (according to the Shapiro-Wilk test and visual inspection), the Kruskal-Wallis test with Bonferroni-adjusted and *post hoc* analyses was used to compare *CG*, *PwH*_*minor*_, and *PwH*_*major*_. Effect sizes for pairwise comparisons were calculated as Cohen *r* and categorized as small (*r* < 0.30), medium (*r* = 0.30-0.50), or large (*r* > 0.50).

A correlation analysis was further carried out in PwH to examine possible associations between the strength tests and joint health with the individual muscles in the relaxed and contracted state as well as with the *SIR*. The 2-tailed Spearman rank correlation coefficient was used. Correlation coefficients (*r*_*s*_) are presented and can be interpreted using the same threshold criteria (small: *r*_*s*_ < 0.30, medium: *r*_*s*_ = 0.30-0.50, large: *r*_*s*_ > 0.50). For all tests, a significance level of *P* ≤ .05 with a 95% CI was set.

## 3 Results

A total of 40 PwH and 20 healthy controls of similar age and BMI were included in this study. PwH were divided into *PwH*_*minor*_ and *PwH*_*major*_ as described above. Table 1 shows the anthropometric data, joint status, and medical history of the participants. All participants were able to complete the tests successfully, and no adverse events occurred. Results are presented for the total group (*PwH*_*total*_). Moreover, to take into account the heterogeneity of the PwH, this study focuses on comparing the *PwH*_*minor*_, *PwH*_*major*_, and *CG* groups. To evaluate overall muscle mechanical properties across all participants without subgroup dichotomization, the total hemophilia cohort (*PwH*_*total*_) was compared with the healthy *CG*, as illustrated in Figure 2. Results of myotonometry performed in the relaxed state (*S*_*rel*_) reveal that stiffness differed depending on the muscles and joint affectedness. For RF, VM, VL, GM, and GL muscles, a significantly higher stiffness was observed in *PwH*_*major*_ compared with the *CG* (all *P* ≤ .05, effect sizes ranging from *r* = 0.446 to 1.01). Further, *PwH*_*major*_ exhibited significantly higher stiffness than *PwH*_*minor*_ in these muscles (all *P* ≤ .05, effect sizes ranging from *r* = 0.397 to 0.882). No difference was observed between *PwH*_*minor*_ and *CG* in any muscle (Figure 3A; detailed medians, IQRs, exact *P* values, and effect sizes for all muscles are provided in the Supplementary Table).

**Table 1:** Presentation and comparison of anthropometric and hemophilia-specific data between *PwH*_*total*_ and *CG* = *P* value 1 according to the Mann-Whitney U-test and between *PwH*_*minor*_ (lower half based on the HJHS), *PwH*_*major*_ (upper half based on the HJHS), and *CG* = *P* value 2 according to the Kruskal-Wallis test.

Parameter	*PwH*_*total*_ (*n* = 40)	*CG* (*n* = 20)	*P* value 1 (effect size)	*PwH*_*minor*_ (*n* = 20)	*PwH*_*major*_ (*n* = 20)	*P* value 2 (effect size)
**Ethnicity/nationality**	White/German (100%)	White/German (100%)				
**Age (y)**	56.0 [47.25-60.75] (19.0-76.0)	52.5 [39.5-62.0] (24.0-69.0)	.68 (0.053)	49.0 [39.0-56.8] (19.0-62.0)^a^	59.5 [56.0-64.8] (26.0-76.0)	.005 (0.511)
**Body mass (kg)**	80.5 [72.5-90.0] (62.0-130.0)	83.5 [80.3-94.5] (71.0-118.0)	.09 (0.218)	79.5 [72.0-89.0] (63.0-130.0)	82.0 [74.3-93.3] (62.0-112.0)	.21
**Height (m)**	1.78 [1.75-1.85] (1.65-1.96)	1.81 [1.77-1.89] (1.70-1.92)	.12 (0.203)	1.79 [1.76-1.85] (1.69-1.96)	1.78 [1.75-1.85] (1.65-1.91)	.21
**Body mass index (kg/m^2^)**	25.2 [23.4-27.7] (21.3-33.8)	25.8 [23.7-27.5] (22.2-37.7)	.42 (0.105)	24.6 [23.0-26.3] (21.3-33.8)	25.8 [23.4-29.0] (21.5-30.7)	.41
**HJHS global (score points)**	33.0 [18.5-49.75] (1.0-69.0)^b^	3.5 [2.3-9.8] (2.0-17.0)	<.001 (0.673)	19.0 [10.3-22.8] (1.0-36.0)^a^^,^^b^	49.5 [42.3-58.8] (29.0-69.0)^b^	<.001 (1.045)^b^ .047 (0.383)^a^
**HJHS sum lower limbs (score points)**	24.0 [11.0-34.0] (0.0-52.0)^b^	3.0 [2.0-6.0] (0.0-14.0)	<.001 (0.883)	11.0 [5.3-16.5] (0.0-24.0)^a^^,^^b^	34.0 [30.3-39.8] (25.0-52.0)^b^	<.001 (1.418)^b^ .01 (0.456)^a^
**HEAD-US global (score points)**	9.0 [5.0-16.0] (0.0-24.0)			5.0 [2.5-8.8] (0.0-16.0) a	13.0 [12.0-19.0] (4.0-24.0)	<.001^c^ (0.675)
**HEAD-US sum lower limbs (score points)**	5.0 [1.0-9.0] (0.0-19.0)			3.5 [1.0-5.0] (0.0-12.0) a	8.0 [5.0-13.0] (0.0-19.0)	.003^c^ (0.460)
**Most affected joints (*n*)**	Ankle: 9 left/7 right Knee: 4 left/2 right Elbow: 7 left/3 right Other: 2 None: 6			Ankle: 4 left/3 right Knee: 1 left/0 right Elbow: 4 left/2 right Other: 1 None: 5	Ankle: 5 left/4 right Knee: 3 left/2 right Elbow: 3 left/1 right Other: 1 None: 1	
**Type of hemophilia (*n*)**	Mild: *n* = 2 Moderate: *n* = 8 Severe: *n* = 30 A: *n* = 32 B: *n* = 8			Mild: *n* = 2 Moderate: *n* = 6 Severe: *n* = 12 A: *n* = 16 B: *n* = 4	Mild: *n* = 0 Moderate: *n* = 2 Severe: *n* = 18 A: *n* = 16 B: *n* = 4	
**Factor treatment (prophylaxis/on demand)**	Prophylaxis: 36 On demand: 4			Prophylaxis: 16 On demand: 4	Prophylaxis: 20 On demand: 0	
**ABR self-reported**	1.0 [0.0-2.0] (0.0-17.0)			1.0 [0.0-2.0] (0.0-7.0)	0.5 [0.0-2.5] (0.0-17.0)	.57^c^ (0.090)
**HIV (*n*)**	Yes: 6/no: 34			Yes: 1/no: 19	Yes: 5/no: 15	
**Hepatitis (*n*)**	A: 1; C: 3; B, C: 1; A, B, C: 1; none: 34			A: 1; C: 1; A, B, C: 1; none: 17	C: 2; B, C: 1; none: 17	

Data presented as median [IQR, 25th-75th percentile] (min-max).

CG, control group; HEAD-US, Hemophilia Early Arthropathy Detection with Ultrasound; HJHS, Hemophilia Joint Health Score; PwH, people with hemophilia.

a *P* < .05 vs *PwH*_*major.*_

b *P* < .05 vs *CG*.

c Use of the Mann-Whitney U-test due to the lack of a HEAD-US in *CG*.

**Figure 2:**
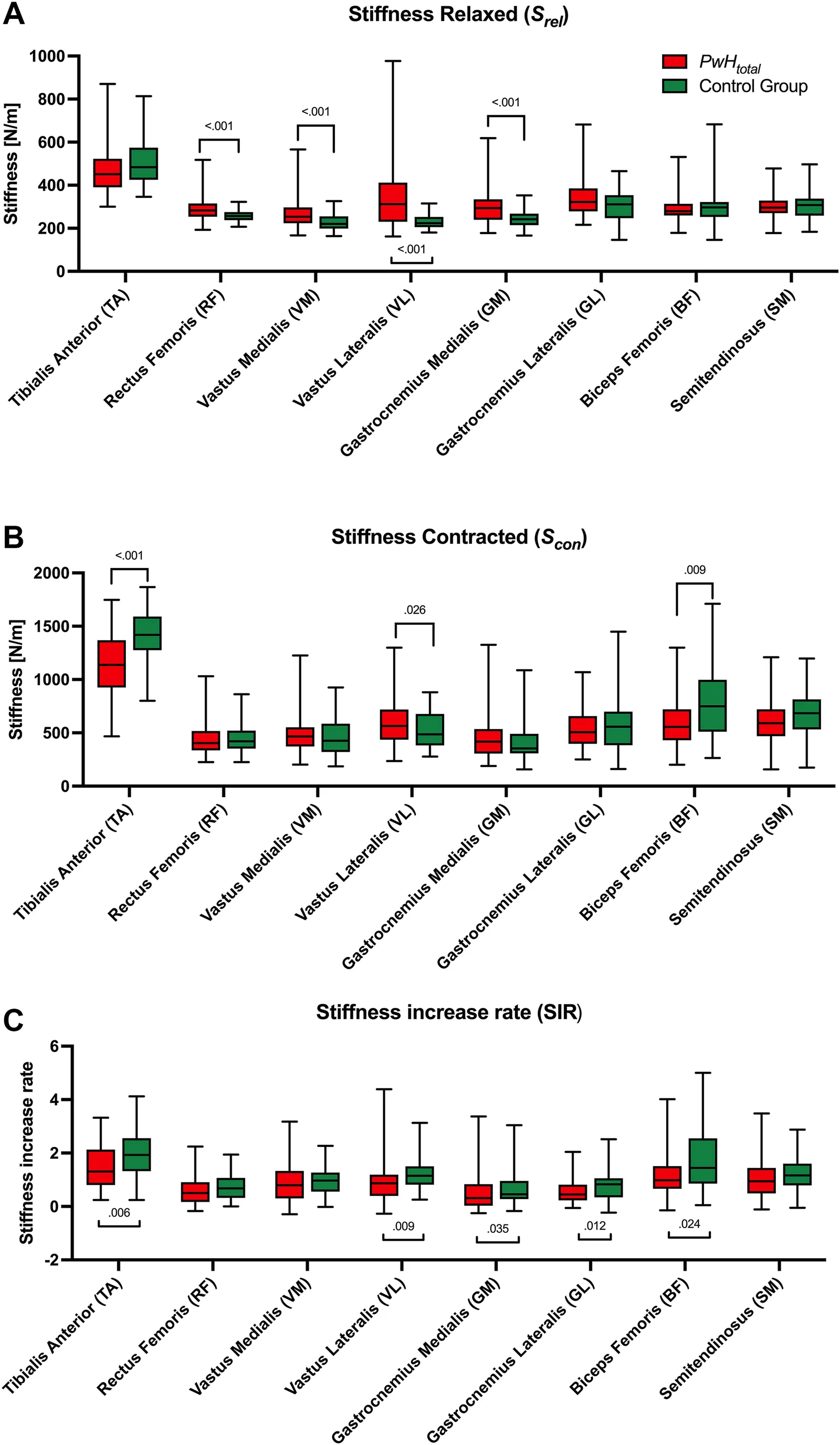
Comparison of myotonometric parameters between the total hemophilia cohort (*PwH*_*total*_) and the healthy control group (*CG*). (A) Dynamic muscle stiffness in the relaxed state (*S*_*rel*_), (B) dynamic muscle stiffness in the contracted state (*S*_*con*_), (C) stiffness increase rate (*SIR*). Data are presented as boxplots displaying the median, IQR, and full data range from minimum to maximum.

**Figure 3:**
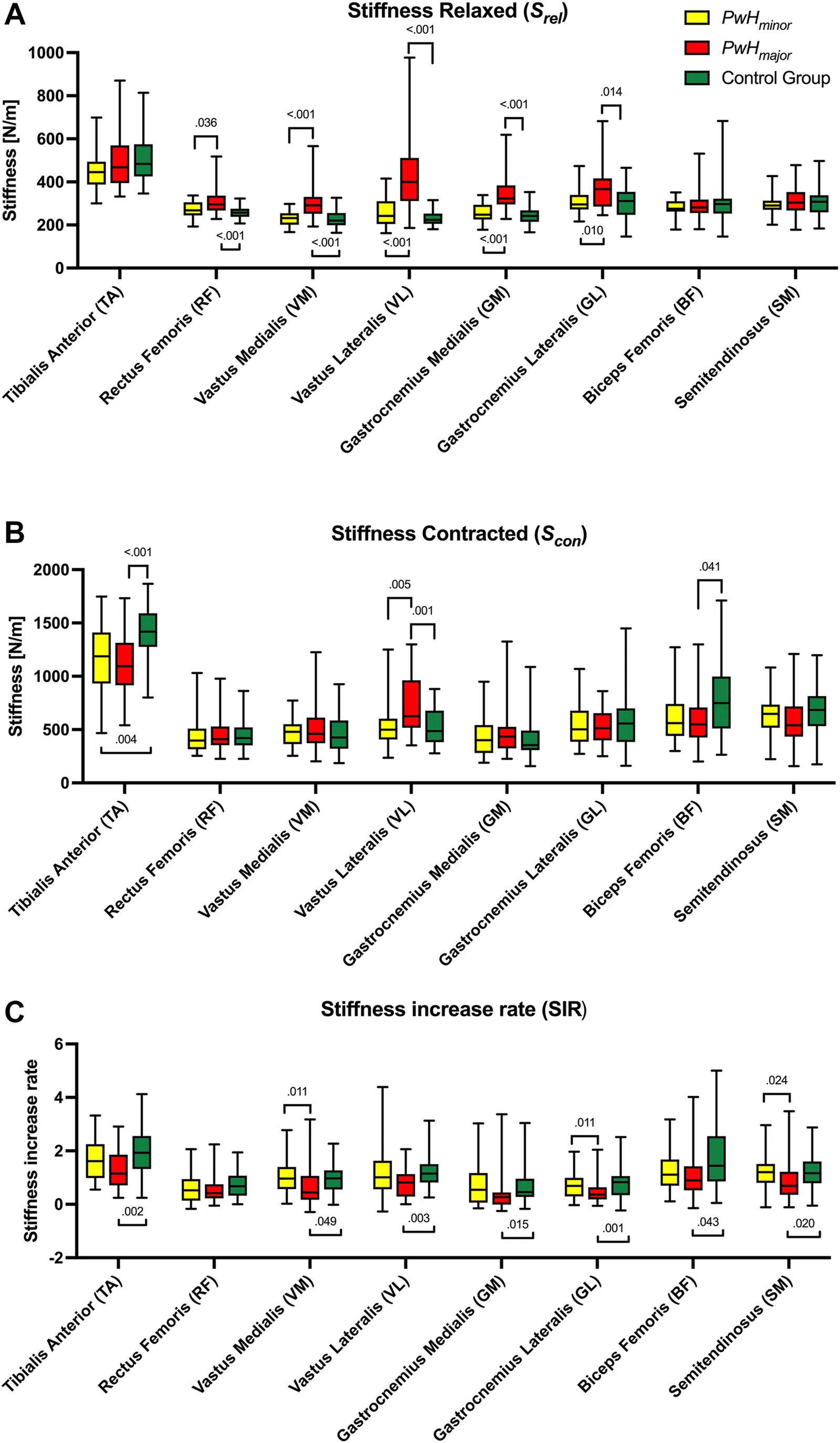
Comparison of myotonometric parameters between the *PwH*_*minor*_, *PwH*_*major*_, and the healthy control group (*CG*). (A) Dynamic muscle stiffness in the relaxed state (*S*_*rel*_), (B) dynamic muscle stiffness in the contracted state (*S*_*con*_), and (C) stiffness increase rate (*SIR*). Data are presented as boxplots displaying the median, IQR, and full data range from minimum to maximum. HJHS, Hemophilia Joint Health Score; *PwH*_*minor*_, people with hemophilia with minor joint impairment based on lower limb HJHS; *PwH*_*major*_, people with hemophilia with major joint impairment based on lower limb HJHS. Detailed quantitative values are provided in the Supplementary Table.

In the contracted state (*S*_*con*_), in which the absolute stiffness was measured under maximum voluntary muscle contraction, results reveal significantly lower stiffness in the TA muscle in *PwH*_*major*_ and *PwH*_*minor*_ compared with the *CG* (*P* ≤ .05, effect sizes ranging from *r* = 0.511 to 0.683). *PwH*_*major*_ showed significantly less stiffness in the BF muscle than the *CG* (*P* = .04, *r* = 0.390). Conversely, *PwH*_*major*_ showed significantly higher stiffness in the VL muscle than the *CG* and *PwH*_*minor*_ (*P* ≤ .05, effect sizes ranging from *r* = 0.500 to 0.555) (Figure 3B; detailed medians, IQRs, exact *P* values, and effect sizes for all muscles are provided in the Supplementary Table).

Regarding the *SIR*, which represents the relative increase in stiffness due to voluntary contraction (calculated based on *S*_*rel*_ and *S*_*con*_), *PwH*_*major*_ exhibited significantly lower *SIR* than the *CG* in all muscles except the RF (all *P* ≤ .05, effect sizes ranging from *r* = 0.380 to 0.573). Furthermore, *PwH*_*major*_ had a significantly lower SIR than *PwH*_*minor*_ in the VM, GL, and SM muscles (all *P* ≤ .05, effect sizes ranging from *r* = 0.419 to 0.460) (Figure 3C; detailed medians, IQRs, exact *P* values, and effect sizes for all muscles are provided in the Supplementary Table).

Results of the strength tests reveal that *PwH*_*major*_ are weaker in hand grip strength, knee extension, and knee flexion than the *CG* (all *P* ≤ .05, effect sizes ranging from *r* = 0.508 to 0.685) and in some cases also weaker than *PwH*_*minor*_ (all *P* ≤ .05, effect sizes ranging from *r* = 0.379 to 0.386) (Table 2). Correlation analyses of the strength outcomes with the results of myotonometry in the relaxed state reveal that there are weak to moderate negative correlations for the RF (*r*_*s*_ = –0.220), VM (*r*_*s*_ = –0.430), VL (*r*_*s*_ = –0.420), and GM (*r*_*s*_ = –0.272) muscles. In contrast, the HJHS of the legs correlates positively with stiffness of the RF (*r*_*s*_ = 0.309), VM (*r*_*s*_ = 0.656), VL (*r*_*s*_ = 0.683), GM (*r*_*s*_ = 0.539), and GL (*r*_*s*_ = 0.248) muscles (Figure 4A). In the contracted state, positive weak to moderate correlations between the strength tests and the TA (*r*_*s*_ = 0.418), RF (*r*_*s*_ = 0.328), and GL (*r*_*s*_ = 0.309) muscles as well as between the HJHS of the legs and VL (*r*_*s*_ = 0.318) are observed. In contrast, the TA (*r*_*s*_ = –0.169), VM (*r*_*s*_ = –0.081), GM (*r*_*s*_ = –0.033), GL (*r*_*s*_ = –0.074), BF (*r*_*s*_ = –0.090), and SM (*r*_*s*_ = –0.177) muscles are negatively but not significantly correlated with the HJHS of the legs (Figure 4B). For *SIR*, the strength tests are positively correlated with the TA (*r*_*s*_ = 0.424), RF (*r*_*s*_ = 0.427), VM (*r*_*s*_ = 0.376), VL (*r*_*s*_ = 0.449), and GL (*r*_*s*_ = 0.402) muscles. In addition, the HJHS is negatively correlated with the TA (*r*_*s*_ = –0.247), VM (*r*_*s*_ = –0.444), VL (*r*_*s*_ = –0.401), GM (*r*_*s*_ = –0.278), and GL (*r*_*s*_ = –0.346) muscles (Figure 4C).

**Table 2:** Comparison of the results of the strength tests between *PwH*_*total*_ and *CG* = *P* value 1 according to the Mann-Whitney U-test and between *PwH*_*minor*_ (lower half based on the HJHS), *PwH*_*major*_ (upper half based on the HJHS), and *CG* = *P* value 2 according to the Kruskal-Wallis test.

Parameter	Side	*PwH*_*total*_ (n = 40)	*CG* (*n* = 20)	*P* value 1 (effect size)	*PwH*_*minor*_ (*n* = 20)	*PwH*_*major*_ (*n* = 20)	*P* value 2 (effect size)
**Hand grip strength (kg)**	Right	34.3 [28.8-43.5] (10.7-62.0)^a^	42.2 [36.6-50.3] (28.7-54.7)	.009 (0.336)	38.2 [31.0-46.3] (20.7-50.3)	33.0 [26.2-39.9] (10.7-62.0)^a^	.005 (0.508)
	Left	34.0 [30.0-41.3] (17.0-61.3)	38.8 [34.9-42.9] (20.7-54.3)	.13 (0.195)	35.5 [29.4-45.7] (20.3-48.0)	34.0 [30.0-40.3] (17.0-61.3)	.27 (0.252)
**Knee extension (*N*)**	Right	293.0 [162.7-408.8] (54.6-607.4)^a^	452.1 [393.2-529.3] (183.7-827.1)	<.001 (0.463)	322.0 [249.2-488.2] (69.9-607.4)^b^	226.3 [129.9-334.4] (54.6-445.5)^a^	<.001 (0.685)^a^; .04 (0.386)^b^
	Left	277.8 [171.3-368.5] (84.1-526.6)^a^	399.4 [353.1-502.9] (201.3-760.7)	<.001 (0.449)	343.4 [227.2-469.9] (84.1-526.6)	218.7 [142.4-299.4] (114.2-465.5)^a^	<.001 (.63)
**Knee flexion (*N*)**	Right	254.2 [169.6-314.8] (91.3-405.6)	273.1 [228.2-349.4] (147.5-472.6)	.27 (0.143)	287.0 [246.6-366.3] (91.3-405.6)^b^	209.0 [162.5-268.8] (100.5-382.2)	.03 (0.379)
	Left	251.4 [194.3-324.1] (67.0-501.3)	237.8 [230.1-333.2] (230.1-333.2)	.52 (0.083)	288.6 [211.6-392.5] (112.8-501.3)	211.9 [166.8-296.1] (67.0-445.8)	.09 (0.331)

Data presented as median [IQR, 25th-75th percentile] (min-max).

CG, control group; HJHS, Hemophilia Joint Health Score; PwH, people with hemophilia.

a *P* < .05 vs *CG*.

b *P* < .05 vs *PwH*_*major*_.

**Figure 4:**
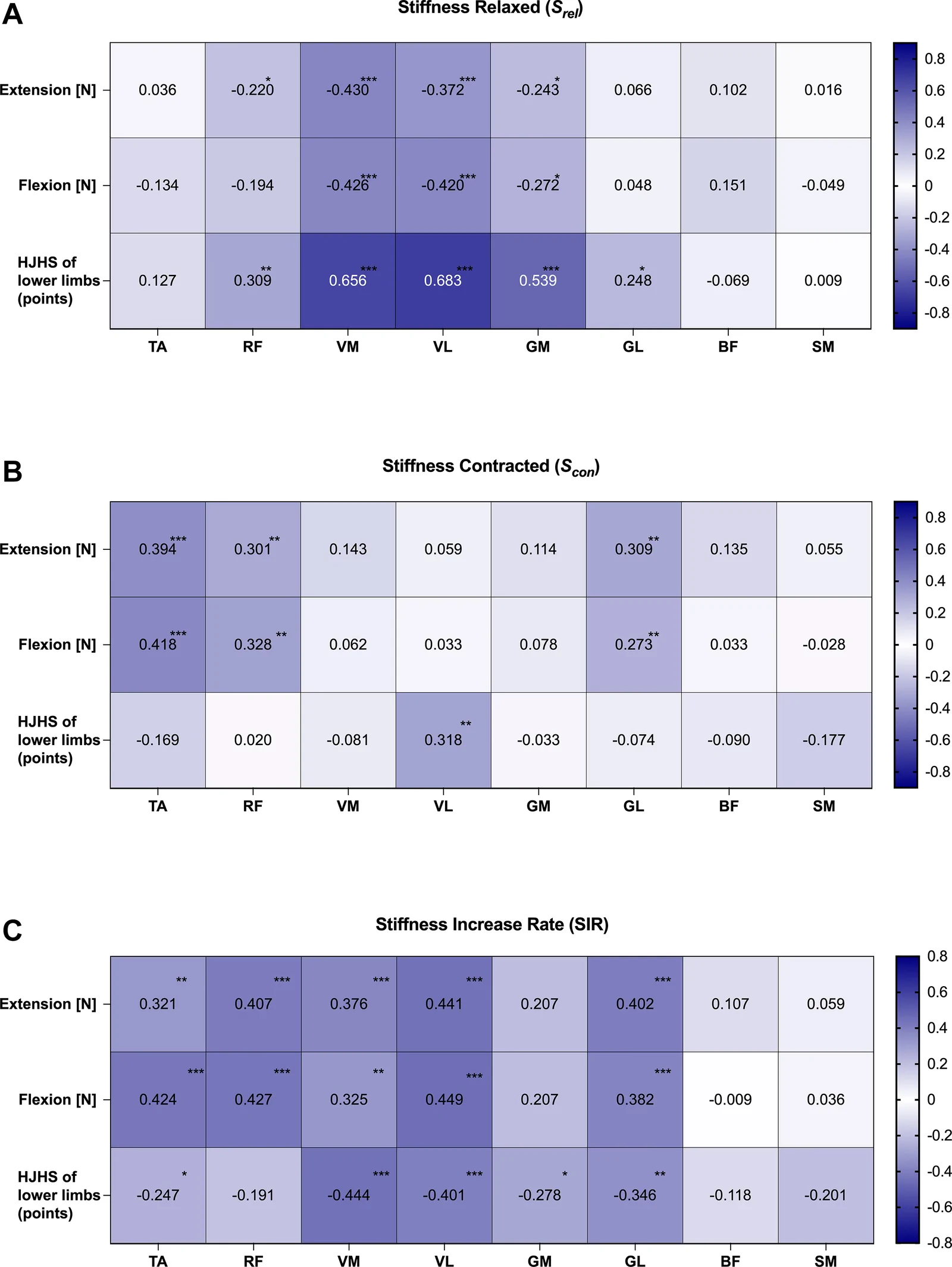
Correlogram displaying Spearman rank correlation coefficients ($r_{s}$) between clinical joint health, isometric muscle strength, and myotonometric muscle parameters in people with hemophilia. Correlations are presented separately for (A) relaxed state (*S*_*rel*_), (B) contracted state (*S*_*con*_), and (C) stiffness increase rate (*SIR*). Color intensity and numerical values within each tile represent the magnitude and direction of correlation coefficients (*r*_*s*_). BF, biceps femoris; GL, gastrocnemius lateralis; GM, gastrocnemius medialis; HJHS, Hemophilia Joint Health Score; RF, rectus femoris; SM, semitendinosus; TA, tibialis anterior; VL, vastus lateralis; VM, vastus medialis.

## 4 Discussion

The aim of this study was to investigate the biomechanical viscoelastic muscle properties of PwH in both relaxed and contracted states and their relationship with muscle strength. Specifically, the study examined (1) whether muscle stiffness in a relaxed state differs between PwH with varying levels of HA severity and matched healthy controls, (2) how muscle properties are altered in PwH compared with *CG* under voluntary contraction, and (3) the extent to which these biomechanical properties are associated with isometric muscle strength.

The data presented herein for the first time show different manifestations of biomechanical viscoelastic muscle properties (ie, stiffness) depending on the progression of HA in different lower extremity muscle groups using a direct noninvasive measurement in adult PwH. In particular, *S*_*rel*_ was significantly higher in PwH with advanced HA (*PwH*_*major*_) compared with those with less advanced HA (*PwH*_*minor*_) and *CG*. This increase was observed in the anterior thigh muscles RF, VM, and VL and the calf muscles GM and GL. In contrast, TA and the posterior thigh muscles BF and SM showed comparable stiffness values across all groups. This observed increase in passive stiffness aligns with findings from neuromuscular and clinical populations where structural tissue remodeling and altered muscle tone leading to elevated resting stiffness have been documented using myotonometry [12], [13], [14].

As there are no established normative values for muscle stiffness using myotonometry in general populations, interpretation relies on comparison with the *CG* and related literature. This increase in *S*_*rel*_ in *PwH*_*major*_ aligns with hemophilia-specific evidence demonstrating structural and neuromuscular remodeling in response to chronic joint disease. Recurrent hemarthroses lead to arthrogenic muscle inhibition, chronic low-grade inflammation, and secondary muscular atrophy [3], [7]. Hemophilia-specific morphologic studies have confirmed reductions in muscle cross-sectional area as well as altered pennation angles and fascicle lengths in affected limbs [6]. These structural alterations, combined with altered electromyographic activation patterns during weight bearing [8], may potentially contribute to adaptive muscle stiffening as a hypothetical compensation strategy for underlying joint instability. Although OA literature describes similar functional stiffness increases [40], the underlying drivers in PwH may be linked to hemophilia-specific joint and muscle tissue degeneration. Functional differences related to muscle action may also play a role. Although the anterior thigh muscles are mainly responsible for knee extension and joint stabilization, the posterior thigh muscles contribute primarily to knee flexion and offer less stabilization [41]. A potential increase in stabilization demand in HA could offer an exploratory explanation for the observed difference in *S*_*rel*_ between anterior and posterior thigh musculature. However, as joint stability and local muscle architecture were not directly evaluated, this interpretation remains speculative.

Regarding the musculature responsible for the dorsiflexion and plantarflexion of the ankle joint, a distinction was observed between the anterior (TA) and posterior (GM and GL) lower leg muscles. The ankle, often affected by HA, typically shows a reduced range of motion, particularly in dorsiflexion, which also leads to diminished muscle strength [42]. The finding that TA *S*_*rel*_ in PwH was comparable to that in the *CG* is surprising, as joint contractures or restricted ankle range of motion are frequent in severe HA. However, whether resting muscle stiffness directly relates to structural contractures or altered joint mechanics cannot be confirmed without concurrent morphologic imaging. In contrast, the GM and GL that are primarily responsible for plantarflexion demonstrated significantly increased *S*_*rel*_ in *PwH*_*major*_ compared with *CG* and *PwH*_*minor*_. Whether this elevation reflects altered passive tissue properties or an exploratory stabilizing role remains to be clarified in future functional studies.

Under contracted conditions *S*_*con*_, in which stiffness is measured during maximum voluntary isometric contraction, 3 muscles showed statistically significant differences. In TA and BF, *PwH*_*major*_ exhibited lower *S*_*con*_ compared with *CG* and *PwH*_*minor*_, whereas *S*_*con*_ in VL was higher in *PwH*_*major*_ than in the other 2 groups. The only previous study to use myotonometry in young PwH (the study by Gönen et al. [23]) also found altered mechanical properties in the VL muscle of pediatric patients. Although those authors reported reduced elasticity in the VL, defined as the tissue’s ability to return to its original form after deformation, this property is biomechanically associated with increased stiffness. Despite the different study population, this supports the relevance of the VL muscle in PwH. In addition, a study that examined muscle activity in the standing position in adult PwH using surface electromyography also showed increased muscle activity in the anterior thigh and no increased muscle activity in the posterior thigh [8].

When evaluating contracted stiffness (*S*_*con*_), an unexpected discrepancy emerged between the quadriceps and the tibialis anterior. Although *S*_*con*_ was elevated in the VL of *PwH*_*major*_, the tibialis anterior demonstrated a reduced *S*_*con*_ despite showing no difference in *S*_*rel*_. Although this could partially relate to limited ankle dorsiflexion and altered gait mechanics [43], attributing this divergent response solely to functional or stabilizing differences remains speculative. Because local muscle architecture, intramuscular fatty infiltration, muscle fibrosis, and exact joint range of motion were not assessed, muscle-specific morphologic alterations or joint constraints may have contributed to these opposite findings. Consequently, *S*_*con*_ alone is limited in evaluating active muscle contraction capacity, as it does not account for baseline resting stiffness. In this context, the *SIR* represents a clearer and functionally more expected parameter. By expressing the relative stiffness gain from the relaxed to the contracted state, the *SIR* accounts for individual baseline variability in resting tone and provides a more reliable metric of dynamic muscular adaptation. In *PwH*_*major*_, *SIR* was significantly lower across all measured muscles except RF compared with *CG* and, for VM, GL, and SM, even lower than *PwH*_*minor*_. These results may reflect structural and cellular alterations in skeletal muscle that have been associated in the literature with age-related sarcopenia [35]. However, given that no statistically significant age difference existed between *PwH*_*minor*_ and *CG* in this cohort, these changes are likely induced by advanced HA. This aligns with findings from a body composition study involving 201 PwH, which reported that leg muscle mass was significantly lower in patients with severe hemophilia compared with those with moderate or mild forms [44]. This supports the present assumption, as patients with severe hemophilia were statistically more affected by HA, whereas age differences were ruled out.

The altered functional muscular status of PwH was also evident in the strength assessments for knee extension and flexion (Table 2). *PwH*_*major*_ showed reduced strength values on the affected limb compared with both *CG* and *PwH*_*minor*_. These findings are consistent with previous studies [4]. Novel insights emerged from the correlation between myotonometry results and strength tests. Higher strength values were associated with lower *S*_*rel*_ in the anterior thigh and GM muscles, and with higher *S*_*con*_ in TA, RF, and GL (Figure 4A and B). In addition, greater strength correlated with improved *SIR* across all measured muscles except the posterior thigh (Figure 4C). These findings support the relevance of myotonometry for assessing biomechanical viscoelastic properties, as they demonstrate a link between muscle stiffness (both in relaxed and contracted states) and functional strength capacity.

This study provides the first insights into the biomechanical viscoelastic muscle properties in adult PwH using myotonometry. Notably, these biomechanical parameters may provide valuable noninvasive insights into hemophilia-specific muscle pathology. Although OA models offer broader parallels for degenerative joint disease [16], [40], [45], the observed combination of elevated resting stiffness and impaired active contractility in *PwH*_*major*_ may hypothetically reflect underlying hemophilia-specific pathophysiology. It can be hypothesized that chronic joint damage, reduced muscle volume, and altered motor unit recruitment collectively impair dynamic force generation while elevating passive tissue resistance, although direct structural and histologic studies are required to confirm these mechanisms. The fact that PwH with more advanced HA differ significantly from both *CG* and minorly affected PwH in terms of stiffness further underscores the high heterogeneity in joint health within the PwH population, complicating generalizations. Nonetheless, the results indicate that preservation of joint health should be a primary therapeutic goal. Myotonometry to assess muscle stiffness appears to be a promising noninvasive method for assessing the biomechanical viscoelastic properties of individual muscles in PwH. However, this study alone is insufficient to derive therapeutic consequences. Future research should aim to implement targeted physical therapy and exercise interventions for managing HA, supported by comprehensive clinical testing and longitudinal myotonometric assessments to track changes over time.

### 4.1 Practical and methodological implications

From a methodological perspective, our findings demonstrate that absolute stiffness values alone during contraction or rest may be confounded by local baseline variability or structural tissue adaptations. Therefore, researchers and clinicians should prioritize parameters such as the SIR to accurately evaluate dynamic muscular function and joint stabilization capacity. From a practical perspective, the MyotonPRO represents a rapid, noninvasive, and objective point-of-care tool for clinical hemophilia management. In routine physical therapy and medical follow-up, bedside myotonometric assessment allows for early identification of subtle functional deficits before severe structural arthropathy progresses. This objective data can assist clinicians in evaluating individual muscle health, monitoring treatment response, and prescribing targeted exercise programs to enhance functional joint protection in PwH.

### 4.2 Limitations

There are some limitations that should be considered to adequately interpret the results presented. The cross-sectional design precludes any causal inferences regarding the observed associations, and the absence of longitudinal data limits the ability to assess changes in muscle stiffness and function over time. Further, the heterogeneity within the PwH cohort may have introduced variability that could affect the generalizability of the findings. Yet, PwH were stratified based on the joint status to provide an estimate of joint affectedness on lower extremity muscle stiffness. A notable limitation is that the MyotonPRO device is influenced by subcutaneous adipose tissue. Although BMI was recorded, we did not objectively quantify fat thickness via ultrasound [46]. Furthermore, strict standardization of the joint range of motion was challenging. Because of severe HA, some patients were unable to assume the predefined joint positions required for the measurements, specifically for knee flexion and ankle dorsiflexion. These necessary protocol adaptations introduce a potential measurement bias, as muscle and tendon stiffness are highly dependent on exact joint positioning and muscle length [32]. However, these adjustments were essential to include patients with severe contractures. Excluding this subgroup would have caused a significant selection bias and failed to reflect the clinical reality of this population. Finally, recruiting participants at specific hemophilia events might have led to a biased sample. These individuals may represent a more health-conscious or socially active subgroup, potentially limiting the generalizability of our findings to the broader patient population.

## 5 Conclusion

This study provides the first detailed insights into the biomechanical viscoelastic muscle properties of adult PwH using noninvasive myotonometry. The findings reveal that PwH with advanced HA exhibit increased muscle stiffness at rest *S*_*rel*_ and reduced stiffness during maximal voluntary contraction *S*_*con*_, particularly in muscle groups responsible for stabilizing the commonly affected knee and ankle joints. The differences observed in the *SIR* between *PwH*_*major*_, *PwH*_*minor*_, and *CG* suggest structural and functional alterations in skeletal muscle associated with the severity of HA. Furthermore, the correlations between myotonometric parameters and muscle strength underscore the functional relevance of these biomechanical properties, highlighting a progressive loss of muscle function in PwH with advanced joint damage.

However, given the observational cross-sectional design of this study, causal relationships cannot be established, and statements regarding direct clinical implementation must be made with caution. Future longitudinal and interventional research is required to determine whether myotonometric monitoring can effectively guide clinical decision-making or individual therapy planning.
