# Supplementary material for: Biomechanical viscoelastic properties in leg muscles of people with hemophilia: a case control study
Source: Res Pract Thromb Haemost. 2026 Oct 1;10(7):107000. doi: 10.1016/j.rpth.2026.107000 (PMC13734919; doi:10.1016/j.rpth.2026.107000)
Supplement: Supplementary file 1 [file mmc1.docx]

**Supplement 1:** Presentation and comparison of myotonometric muscle stiffness in the (A) relaxed state (*S_rel_*), (B) contracted state (*S_con_*), and (C) stiffness increase rate (*SIR*) between *PwH_major_*, *PwH_minor_*, and *CG*. Data are presented as median and [IQR, 25th to 75th percentile] (min-max). Stiffness values are reported in N/m. Overall group differences across the three cohorts were analyzed using the non parametric Kruskal Wallis test. Pairwise post hoc comparisons between individual groups were evaluated using the Mann Whitney U test with Bonferroni correction for multiple testing. Effect sizes for pairwise post hoc comparisons are reported as Cohen r values. *CG* = healthy control group; PwH = people with haemophilia; *PwH_minor_* = people with haemophilia with minor joint affectedness (lower half based on HJHS); *PwH_major_* = people with haemophilia with major joint affectedness (upper half based on HJHS); IQR = interquartile range; TA = tibialis anterior muscle; RF = rectus femoris muscle; VM = vastus medialis muscle; VL = vastus lateralis muscle; GM = gastrocnemius medialis muscle; GL = gastrocnemius lateralis muscle; BF = biceps femoris muscle; SM = semitendinosus muscle.

| **Muscle** | ***PwH_minor_* (n=20)** | ***PwH_major_***  **(n=20)** | ***CG***  **(n=20)** | **Kruskal Wallis p value** | ***PwH_major_* vs**  ***CG***  **p value (effect size)** | ***PwH_major_* vs *PwH_minor_***  **p value (effect size)** | ***PwH_minor_* vs *CG***  **p value (effect size)** |
| --- | --- | --- | --- | --- | --- | --- | --- |
| **(A) *S_rel_* (N/m)** | | | | | | | |
| **TA** | 445.0  [387.8-493.5]  (300.0-699.0) | 467.5  [394.3-569.3]  (332.0-870.0) | 484.1  [425.3-574.5]  (346.0-814.0) | .138 |  |  |  |
| **RF** | 268.0  [244.5-305.8]  (193.0-337.0) | 295.5  [267.0-336.5]  (228.0-518.0) | 256.9  [238.0-275.8]  (207.0-323.0) | <.001 | .001 (.718) | .036 (.397) | .128 (.320) |
| **VM** | 232.0  [201.5-254.3]  (167.0-298.0) | 291.5  [252.0-330.5]  (193.0-566.0) | 220.0  [198.8-255.3]  (164.0-326.0) | <.001 | .001 (.876) | .001 (.773) | 1.000 (.104) |
| **VL** | 242.0  [204.5-310.3]  (162.0-415.0) | 399.5  [311.3-511.5]  (186.0-977.0) | 223.4  [205.3-253.3]  (180.0-315.0) | <.001 | .001 (1.010) | .001 (.781) | .141 (.233) |
| **GM** | 248.0  [225.5-294.0]  (178.0-339.0) | 323.0  [295.8-383.5]  (228.0-619.0) | 242.0  [214.3-267.8]  (166.0-353.0) | <.001 | .001 (1.000) | .001 (.882) | 1.000 (.118) |
| **GL** | 295.0  [271.8-339.3]  (216.0-474.0) | 366.0  [285.0-416.3]  (245.0-682.0) | 311.2  [246.8-354.0]  (146.0-465.0) | .004 | .014 (.446) | .010 (.465) | 1.000 (.019) |
| **BF** | 275.5  [262.0-310.8]  (179.0-351.0) | 281.5  [255.0-317.8]  (180.0-531.0) | 297.3  [253.3-321.8]  (146.0-683.0) | .545 |  |  |  |
| **SM** | 290.5  [270.8-313.3]  (201.0-427.0) | 540.0  [434.8-717.3]  (158.0-1209.0) | 308.2  [258.5-338.3]  (184.0-497.0) | .315 |  |  |  |
| **(B) *S_con_* (N/m)** | | | | | | | |
| **TA** | 1187.0  [931.8-1411.3]  (467.0-1748.0) | 1094.5  [916.5-1314.8]  (540.0-1733.0) | 1419.4  [1276.5-1590.5]  (801.0-1868.0) | <.001 | .001 (.683) | .828 (.172) | .004 (.511) |
| **RF** | 398.0  [315.3-509.3]  (254.0-1030.0) | 410.5  [354.3-529.8]  (225.0-978.0) | 420.5  [352.5-521.0]  (226.0-863.0) | .539 |  |  |  |
| **VM** | 478.5  [363.5-551.0]  (254.0-773.0) | 461.0  [372.3-613.0]  (202.0-1226.0) | 426.5  [320.8-585.5]  (186.0-926.0) | .476 |  |  |  |
| **VL** | 499.5  [407.3-601.8]  (235.0-1251.0) | 626.0  [516.8-963.0]  (353.0-1299.0) | 486.5  [382.3-677.0]  (278.0-880.0) | <.001 | .001 (.555) | .005 (.500) | 1.000 (.055) |
| **GM** | 400.5  [280.3-542.8]  (189.0-949.0) | 433.5  [324.8-526.5]  (226.0-1325.0) | 353.5  [308.3-490.8]  (158.0-1088.0) | .377 |  |  |  |
| **GL** | 502.5  [386.0-677.0]  (272.0-1069.0) | 512.5  [399.3-654.3]  (250.0-861.0) | 557.1  [384.0-698.8]  (161.0-1449.0) | .708 |  |  |  |
| **BF** | 561.5  [441.5-741.3]  (299.0-1272.0) | 549.5  [425.0-706.8]  (201.0-1299.0) | 749.0  [510.0-997.8]  (264.0-1711.0) | .031 | .041 (.390) | 1.000 (.068) | .125 (.322) |
| **SM** | 648.0  [517.0-733.8]  (222.0-1083.0) | 304.0  [267.0-353.3]  (178.0-478.0) | 684.0  [532.8-814.8]  (174.0-1198.0) | .056 |  |  |  |
| **(C) *Stiffness increase rate*** | | | | | | | |
| **TA** | 1.62  [0.98-2.25]  (0.55-3.32) | 1.15  [0.71-1.86]  (0.24-2.91) | 1.93  [1.32-2.56]  (0.24-4.12) | .003 | .002 (.532) | .143 (.313) | .501 (.219) |
| **RF** | 0.52  [0.14-0.95]  (-0.17-2.06) | 0.42  [0.22-0.75]  (-0.05-2.24) | 0.68  [0.33-1.07]  (0.00-1.94) | .155 |  |  |  |
| **VM** | 0.97  [0.57-1.40]  (0.02-2.78) | 0.45  [0.17-1.07]  (-0.29-3.18) | 0.97  [0.56-1.27]  (-0.02-2.27) | .008 | .049 (.380) | .011 (.459) | 1.000 (.079) |
| **VL** | 1.01  [0.57-1.63]  (-0.27-4.39) | 0.82  [0.29-1.13]  (0.00-2.06) | 1.15  [0.82-1.51]  (0.26-3.13) | .003 | .003 (.527) | .104 (.334) | .666 (.193) |
| **GM** | 0.55  [0.07-1.17]  (-0.15-3.03) | 0.27  [-0.01-0.44]  (-0.25-3.37) | 0.46  [0.28-0.96]  (-0.17-3.04) | .016 | .015 (.445) | .148 (.311) | 1.000 (.134) |
| **GL** | 0.69  [0.30-0.99]  (-0.03-1.97) | 0.37  [0.18-0.64]  (-0.06-2.04) | 0.83  [0.34-1.06]  (-0.23-2.52) | <.001 | .001 (.573) | .011 (.460) | 1.00 (.112) |
| **BF** | 1.11  [0.70-1.68]  (0.11-3.18) | 0.90  [0.52-1.42]  (-0.14-4.02) | 1.45  [0.86-2.55]  (0.05-5.00) | .048 | .043 (.388) | .952 (.158) | .439 (.230) |
| **SM** | 1.20  [0.81-1.51]  (-0.11-2.96) | 0.69  [0.36-1.22]  (-0.11-3.48) | 1.17  [0.79-1.60]  (-0.05-2.88) | .008 | .020 (.430) | .024 (.419) | 1.000 (.010) |
